# Artificial Intelligence in Skin Diseases: Fulfilling its Potentials to Meet the Real Needs in Dermatology Practice

**DOI:** 10.34133/2022/9791467

**Published:** 2022-08-08

**Authors:** Yicen Yan, Shenda Hong, Wensheng Zhang, Hang Li

**Affiliations:** ^1^Department of Dermatology and Venereology, Peking University First Hospital, No. 8 Xishiku St., Xicheng District, Beijing 100034, China; ^2^National Clinical Research Center for Skin and Immune Diseases, No. 8 Xishiku St., Xicheng District, Beijing 100034China; ^3^Beijing Key Laboratory of Molecular Diagnosis on Dermatoses, No. 8 Xishiku St., Xicheng District, Beijing 100034China; ^4^NMPA Key Laboratory for Quality Control and Evaluation of Cosmetics, No. 8 Xishiku St., Xicheng District, Beijing 100034China; ^5^National Institute of Health Data Science, Peking University, No. 38 Xueyuan Rd, Haidian District, Beijing 100191, China; ^6^Institute of Automation, Chinese Academy of Sciences, No. 95 Zhongguancun East Road, Haidian District, Beijing 100190, China

Artificial intelligence (AI) medical image analysis techniques based on deep learning and machine learning have developed rapidly in recent years. Since the diagnosis of skin diseases is mainly based on the morphology of lesions, dermatology is considered a promising area for AI image analysis techniques. In 2017, scientists from Stanford University published a milestone paper in Nature to show the performance of AI was comparable to dermatologists in the classification and recognition of skin cancer, using convolutional neural network (CNN) models trained on nearly 130,000 clinical images [1]. Since then, many countries have been actively developing similar products. So far, the U.S. Food and Drug Administration has approved 3Drem, Google DeepMind, and SkinVision. In China, Youzhi Pifu, VoxelCloud DermX, and Meitueve have entered the public view. The public’s interest in AI roars, and the imagination of AI replacing dermatologists seems to be a reality in the foreseen future. 

However, from the perspective of dermatologists, the clinical application of AI is far from fulfilling its purpose. Developing effective and valid AI tools for dermatologists requires understanding the real-world needs of AI in clinical settings.

## 1. The Unmet Needs in Three Clinical Scenarios for AI Application

The first application scenario is screening for severe dermatoses in primary care. General and family doctors see a large number of patients with dermatoses. But due to the lack of experience and dermatology training, they are not fully competent to identify severe dermatoses such as cutaneous malignancies that must be referred to specialists. Diagnostic assistant tools with high sensitivity and efficiency are necessary to help general practitioners discern cases with significant outcomes if missed. Though high efficiency is the inherent advantage of AI, improving the sensitivity of the algorithm relies on generalizable databases with a full spectrum of dermatoses and patients in different age groups [2].

The second scenario for AI application is to assist hospital dermatologists in improving their diagnostic performance. The skin lesions of many skin diseases are nonspecific, especially in their early stages of diseases, and thus can be easily misdiagnosed even by experienced specialists. In this circumstance, high specificity is the priority demand for AI tools. Additionally, hospital dermatologists need tools that can provide innovative diagnostic methods or reveal new features of lesions. As far as these requirements are concerned, current deep learning models such as CNN have certain limitations. Firstly, developing high-quality diagnostic assistant tools for dermatologists requires abundant training cases, which is limited by the incidence of the specific skin disease. For instance, though malignant melanoma is a hot spot for AI applications, it is not easy to collect the enough malignant melanoma cases in China. Less than 20000 new cases of malignant melanoma are diagnosed nationwide every year [3, 4], far behind the required number to reach satisfactory specificity. Secondly, annotating image is one of the crucial aspects of developing AI-based diagnostic models. Accurate annotation is usually time-consuming and costly since it needs agreement among multiple dermatologists. Such intense clinicians’ expertise demand can explain why clinical data, especially images, are mostly unannotated. Thirdly, there is a lack of understanding of how the diagnosis is predicted by deep learning models (usually called “black box”). The intelligibility generates confidence and trust. Without understanding why an algorithm gives one diagnosis rather than another, dermatologists will not be able to confidently adopt the assisting tools in clinical practice.

The third scenario where AI can play a role is the long-term management of chronic dermatoses. For example, patients undertaking skin malignancy operations should regularly see the doctor to evaluate surgery outcomes. AI can provide the necessary tool for remote consolation to make the follow-up efficiently with a lower cost. Additionally, the early recurrence of tumors is usually atypical with indistinguishable and mild lesions, and accurately identifying them by AI remains to be an unmet clinical need. Well-developed AI tool could make a preliminary assessment of tumor recurrence with deep learning-based image analysis algorithm and notify the patients and dermatologists timely.

AI has great potentials to assist clinicians in providing quality healthcare services for patients with skin diseases and improving the efficiency and accessibility of healthcare. On the other hand, the accuracy of AI assisting tools needs improvements. The lack of interpretability impedes doctors’ and patients’ confidence in the predicted results. A gap exists between the status quo of technology and expectations for safety and reliability derived from the “first, do no harm” principle [5]. To close the gap, the joint efforts of dermatologists and AI scientists are the crux to translating the AI’s healthcare changing potentials into the real benefits for patients.

## 2. Three Aspects to Improve the Performance of AI to Help Dermatology Practice

Efforts can be made to improve the AI-assisted diagnosis systems from various aspects such as data, algorithms, and applications. From a data perspective, it is necessary to establish a multidimensional, multimodal, and standardized database comprising clinical information, lesion images, dermoscopy, and reflectance confocal microscopy results for different dermatoses in different patient populations. The database should be as comprehensive as possible since it works as the foundation for model development and validation. Developing such a database requires transdisciplinary communication between clinicians and AI scientists to clarify the requirements and address the technical obstacles.

Secondly, AI algorithms should further adapt to real-world databases with inadequate or poor-annotated samples. A potential direction for adaption is to include expert knowledge of dermatology in algorithm development. That can also help improve the performance and intelligibility of AI-assisted diagnosis systems. How to better integrate the expert knowledge such as medical history with lesion morphology and auxiliary examination in the model development needs more research.

As for application, we need to systematically design the strategy and process of human (clinicians) machine (AI diagnostic system) cooperation and balance the sensitivity and specificity of AI algorithm to meet the actual needs for the whole life cycle management of skin diseases. AI scientists and dermatologists’ in-depth collaboration and continuing efforts are crucial to build powerful, practical AI systems for skin diseases.

## References

[1] A. Esteva, B. Kuprel, R. A. Novoa, J. Ko, S. M. Swetter, H. M. Blau, and S. Thrun, “Dermatologist-level classification of skin cancer with deep neural networks,” *Nature*, vol. 542, no. 7639, pp. 115–118, 2017 28117445 10.1038/nature21056 PMC8382232

[2] K. C. A. Khanzode, and R. D. Sarode, “Advantages and disadvantages of artificial intelligence and machine learning: a literature review,” *International Journal of Library & Information Science (IJLIS)*, vol. 9, no. 1, p. 3, 2020

[3] R. Bai, H. Huang, M. Li, and M. Chu, “Temporal trends in the incidence and mortality of skin malignant melanoma in China from 1990 to 2019,” *Journal of Oncology*, vol. 2021, –9, 2021 10.1155/2021/9989824 PMC8407983 34475955

[4] Y. Wu, Y. Wang, L. Wang, P. Yin, Y. Lin, and M. Zhou, “Burden of melanoma in China, 1990-2017: findings from the 2017 global burden of disease study,” *International Journal of Cancer*, vol. 147, no. 3, pp. 692–701, 2020 31671209 10.1002/ijc.32764

[5] J. Wiens, S. Saria, M. Sendak, M. Ghassemi, V. X. Liu, F. Doshi-Velez, K. Jung, K. Heller, D. Kale, M. Saeed, P. N. Ossorio, S. Thadaney-Israni, and A. Goldenberg, “Do no harm: a roadmap for responsible machine learning for health care,” *Nature Medicine*, vol. 25, no. 9, pp. 1337–1340, 2019 10.1038/s41591-019-0548-6 31427808

